# Sildenafil Attenuates Hepatocellular Injury after Liver Ischemia Reperfusion in Rats: A Preliminary Study

**DOI:** 10.1155/2014/161942

**Published:** 2014-06-04

**Authors:** Spyridon Savvanis, Constantinos Nastos, Marios-Konstantinos Tasoulis, Nikolaos Papoutsidakis, Maria Demonakou, Iosifina Karmaniolou, Nikolaos Arkadopoulos, Vassilios Smyrniotis, Kassiani Theodoraki

**Affiliations:** ^1^Experimental Surgical Unit, Aretaieion University Hospital, 11528 Athens, Greece; ^2^Second Department of Surgery, Aretaieion University Hospital, 11528 Athens, Greece; ^3^Pathology Department, Sismanoglio General Hospital, 15126 Athens, Greece; ^4^Department of Anesthesiology, Penteli Pediatric Hospital, 15236 Athens, Greece; ^5^Fourth Department of Surgery, Attikon University Hospital, 12462 Athens, Greece; ^6^Department of Anesthesiology, Aretaieion University Hospital, 11528 Athens, Greece

## Abstract

We evaluated the role of sildenafil in a rat liver ischemia-reperfusion model. Forty male rats were randomly allocated in four groups. The sham group underwent midline laparotomy only. In the sildenafil group, sildenafil was administered intraperitoneally 60 minutes before sham laparotomy. In the ischemia-reperfusion (I/R) group, rats were subjected to 45 minutes of hepatic ischemia followed by 120 minutes of reperfusion, while in the sild+I/R group rats were subjected to a similar pattern of I/R after the administration of sildenafil, 60 minutes before ischemia. Two hours after reperfusion, serum levels of alanine aminotransferase (ALT) and aspartate aminotransferase (AST) were measured and histopathological examination of the lobes subjected to ischemia as well as TUNEL staining for apoptotic bodies was performed. Additionally, myeloperoxidase (MPO) activity and the expression of intercellular adhesion molecule-1 (ICAM-1) were analyzed. Serum markers of hepatocellular injury were significantly lower in the sild+I/R group, which also exhibited lower severity of histopathological lesions and fewer apoptotic bodies, as compared to the I/R group. The I/R group showed significantly higher MPO activity and higher expression of ICAM-1, as compared to the sild+I/R group. Use of sildenafil as a preconditioning agent in a rat model of liver I/R exerted a protective effect.

## 1. Introduction

Liver resections under some type of vascular control are currently favored by many surgeons since they can ensure a less hemorrhagic surgical field by taking advantage of liver tolerance to normothermic warm ischemia [1, 2]. Although such maneuvers are invaluable in preventing excessive blood loss and allow the performance of a safer procedure, they are invariably complicated by ischemia/reperfusion (I/R) injury [3]. Moreover, hepatic I/R injury can also occur in various other clinical contexts, including liver transplantation, hypovolemic shock, and low-output syndrome [4, 5]. In particular, ischemia leads to depletion of cellular energy, accumulation of intracellular sodium, calcium, and reactive oxygen species (ROS), and activation of multiple enzyme systems leading to cell damage [3]. The reestablishment of blood flow through reperfusion can aggravate local tissue injury secondary to an ensuing acute inflammatory response. Reperfused tissue is infiltrated by activated polymorphonuclear leukocytes and platelets while further tissue damage is mediated through cytokine production by leukocytes, complement activation, local imbalance in nitric oxide (NO) levels, accumulation of platelet activating factors and endothelial-cell adhesion molecules, and finally formation of free radicals [6, 7]. This overwhelming inflammatory response manifests as vasoconstriction, intravascular hemoconcentration, neutrophil migration and adherence, and platelet aggregation [8–10]. The ensuing microcirculatory failure can finally culminate in hepatocellular apoptosis and necrosis with repercussions for the liver as well as distant organs [11–13].

NO is a key molecule, which is recognized as an important, yet controversial mediator of physiological and pathological processes inherent in I/R injury since it has been shown to have both protective and deleterious effects on cellular functions [14]. It has been shown to act through a variety of second-messenger cascades although the majority of its effects are mediated through cyclic guanosine monophosphate (cGMP), which is in turn catabolised by phosphodiesterase type 5 (PDE5) that converts cGMP into the inactive GMP and terminates its action [15]. NO is synthesized from L-arginine by three isoforms of the NO synthase (NOS), the endothelial synthase (eNOS), the inducible synthase (iNOS), and the neuronal synthase (nNOS) [16]. eNOS is responsible for the production of basal NO, which maintains normal vascular tone. iNOS, contrary to eNOS, is calcium insensitive and is especially induced under oxidative stress conditions, with controversial results regarding its role in ischemia-reperfusion [16, 17]. Neuronal (nNOS) is involved in neural signaling with no participation in the ischemia-reperfusion events [18].

eNOS-derived NO is considered to have a cytoprotective effect in I/R injury with cGMP playing an important role in regulation of intracellular calcium levels and favorable modulation of platelet function with stimulation of relaxation of contractile cells and resulting vasodilatation [17, 19]. Therefore, inhibition of cGMP degradation by PDE5 inhibitors might preserve the cGMP pool, thus promoting the favorable action of NO and eventually attenuating the manifestations of I/R injury.

Sildenafil is a potent selective inhibitor of PDE5 and is widely being used for the treatment of erectile dysfunction in men. It has also been investigated in the context of persistent pulmonary hypertension with satisfactory results [20]. There is evidence that sildenafil is also capable of inducing a preconditioning-like effect in I/R injury of various tissues such as the heart, lung, kidney, and brain [21–24]. In spite of a notable number of studies on pharmacological strategies aiming at attenuating the manifestations of liver I/R injury, literature is scarce regarding the use of sildenafil in this context. Therefore, we designed this experimental study in order to evaluate the effect of sildenafil in a liver I/R rat model by using histopathological and biochemical parameters. In specific, we tested the hypothesis that sildenafil exerts a protective effect on the liver, as this has been evidenced for other tissues subjected to I/R insults.

## 2. Methods

### 2.1. Animals and Experimental Design

This protocol was approved by the Animal Research Committee of the University of Athens and the Committee of Bioethics of Aretaieion Hospital. Handling and care of the animals was in accordance with European guidelines for ethical animal research.

Forty male Wistar Rats weighing 300–350 g were used. The animals were housed in individual cages, at constant temperature conditions (21°C), with alternating 12-hour light/dark cycles. They were also maintained on a standard diet and water* ad libitum*.

The animals were randomly allocated into four groups: a group which received no treatment and underwent midline laparotomy only (sham group, *n* = 10), a group that underwent midline laparotomy only as the sham group after the administration of sildenafil 0.3 mg/kg 60 minutes before the operation (sild+sham group, *n* = 10), a group that was subjected to partial liver ischemia and reperfusion (I/R group, *n* = 10), and a group that underwent liver ischemia and reperfusion after the administration of sildenafil 0.3 mg/kg 60 minutes before the induction of hepatic ischemia (sild+I/R group, *n* = 10).

Sildenafil was administered at a dose of 0.3 mg/kg with an intraperitoneal injection. This dose is the equivalent of the dose used for the management of pulmonary hypertension [25–27].

### 2.2. Surgical Procedure

All animals were anesthetized with intraperitoneal administration of ketamine 100 mg/kg and xylazine 10 mg/kg. The rats had their abdomen clipped off hair and prepared with povidone-iodine solution. All subsequent procedures were performed using aseptic technique with sterile equipment and prostheses. The rats underwent a 4 cm midline abdominal incision through the musculature and peritoneum. Twenty IU/kg of heparin was administered intraperitoneally. After identification of all liver lobes, the portal vein was identified. In the I/R and the sild+I/R groups, the portal vein and hepatic artery were occluded with the use of atraumatic vascular clips, immediately after the bifurcation of the right lateral branch. The clamp was partial, aiming at blocking the portal venous and hepatic arterial blood supply to the median and left lateral lobes of the liver. This yielded approximately 70% of hepatic ischemia, which was maintained for a 45-minute period. We aimed at partial ischemia in order to avoid prolonged blood pooling in the splanchnic bed, which could result in splanchnic congestion and intestinal injury. For this reason, the gut was monitored macroscopically throughout the ischemic period for signs of portal hypertension. At the end of this period, the clamp was removed and portal and arterial blood flow were restored. Reperfusion was confirmed macroscopically by change of the color of the liver. In the sham and the sild+sham groups, only the hepatic pedicle was identified and hepatic vessel clips were not applied.

The ischemic period was followed by two hours of reperfusion. During this period the abdomen was closed in the midline. At the end of the reperfusion period the abdomen was reopened and blood samples were collected from the abdominal aorta. Biopsies were taken from the liver lobes that were subjected to ischemia.

Anesthesia was maintained throughout the experimental period with intraperitoneal administration of ketamine and xylazine, while at the end of the experiment, the animals were sacrificed by exsanguination while on anesthesia.

### 2.3. Liver Function Tests

After collection, blood samples were centrifuged at 4000 rpm for 20 minutes and serum was stored at −70°C until analysis. Alanine aminotransferase (ALT) and aspartate aminotransferase (AST) levels were determined in systemic circulation using the Dimension RXL analyzer (Dade Behring, Dupond, Delaware).

### 2.4. Histopathological Evaluation

The tissue samples from the ischemic liver lobes were fixed in 10% buffered formaldehyde solution, embedded in paraffin, and then cut in 3–5 *μ*m sections. Sections were stained with hematoxylin-eosin under standard histological methods and were evaluated by light microscopy. A pathology scoring system which assessed hepatocellular liver necrosis with a five-point grading scale and inflammatory infiltration with a three-point grading scale was used, as shown in Table 1.

### 2.5. Myeloperoxidase Assay

Tissue samples from the ischemic liver lobes were stained immunohistochemically for myeloperoxidase (MPO). Briefly, formaldehyde-fixed, paraffin-embedded sections were incubated for 30 minutes at room temperature with 1% polyclonal rabbit antihuman myeloperoxidase antibodies, according to the manufacturer's instructions (Dako Pathology Products, Hamburg, Germany). MPO staining was quantified according to the expression of MPO in three optic fields (40x magnification) as shown in Table 2. All microscopic examinations were performed by an expert pathologist, who was unaware of the treatment group.

### 2.6. Measurement of Intercellular Adhesion Molecule-1 (ICAM-1) mRNA Levels

The expression of ICAM-1 was analyzed by reverse transcription-polymerase chain reaction (RT-PCR). Total RNA was isolated from liver tissue using TRIzol reagent (Invitrogen, Carlsbad, CA). and afterwards, 5 *μ*g RNA was used for complementary DNA synthesis. ICAM-1 gene expression was normalized with *β*-actin gene expression. RT-PCR was performed at the following temperatures: for ICAM-1, denaturation at 94°C for 45 sec, primary annealing at 55°C for 30 sec, and primer extension at 72°C for 90 sec and for *β*-actin, 94°C for 1 min, 55°C for 1 min, and 72°C for 2 min, respectively. The sequences of the primer sets were GAT GCT GAC CCT GGA GAG CA and CAG GGA CTT CCC ATC CAC CT for ICAM-1. Those for *β*-actin were TAT GGA ATC CTG TGG CAT CC and ACA GAA GCA ATG CTG TCA CC. Semiquantification of gene expression was performed and mRNA expression of ICAM-1 was presented as a percentage of *β*-actin.

### 2.7. Apoptosis Assay

Specimens 4 *μ* thick were also cut and stained with the TUNEL assay. This assay is performed by labelling the free 3′-OH ends of DNA strand breaks that are produced after DNA fragmentation during apoptosis, with fluorescent nucleotides in an enzymatic labelling method with terminal deoxynucleotidyl transferase. In our study a commercial kit Apoptosis-1,5 (YLEM, Rome, Italy) was used for the TUNEL assay, according to the manufacturer's instructions. Ten random fields were analyzed for each TUNEL-stained tissue sample. All slides were examined by the same pathologist, who was unaware of the treatment group. Data regarding TUNEL staining were expressed as mean ± SD percentage of nuclei containing apoptotic bodies per high-power field.

### 2.8. Statistical Analysis

Variables were tested for normality of distributions with the Kolmogorov-Smirnov test. AST and ALT values and ICAM-1 mRNA levels as a percentage of *β*-actin and percentage of apoptotic bodies were normally distributed and differences between experimental groups were analyzed with one way analysis of variance (ANOVA), followed by the Holm-Sidak test for* post hoc* comparisons between individual groups. Histology and MPO staining scores did not follow normal distribution and differences between experimental groups were analyzed with one Kruskal-Wallis ANOVA on ranks, followed by the Mann-Whitney *U* test for* post hoc* comparisons between individual groups. All calculations were carried out using SPSS 17.0 for Windows. The level of statistical significance was set to *P* < 0.05. Results are expressed as mean ± SD or as median (25th–75th percentiles) depending on normality of distributions.

## 3. Results

### 3.1. Serum AST and ALT Levels

**Figure 1:**
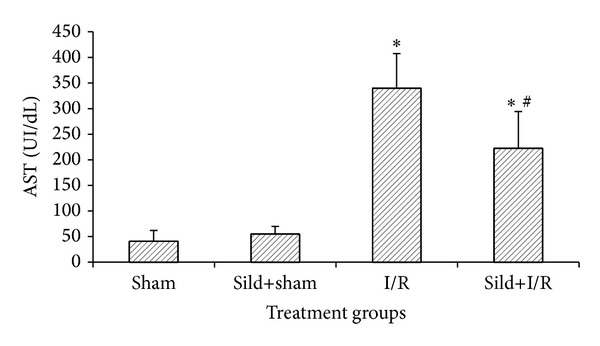
AST serum levels two hours after reperfusion; *P < 0.05 in comparison to the sham group; ^#^ *P* < 0.05 in comparison to the I/R group.

**Figure 2:**
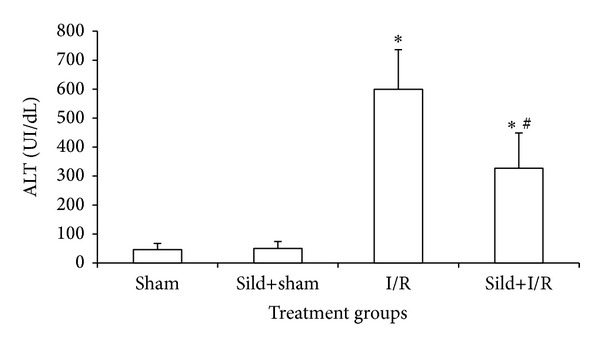
ALT serum levels two hours after reperfusion; *P < 0.05 in comparison to the sham group; ^#^ *P* < 0.05 in comparison to the I/R group.

I/R of the liver substantially increased serologic markers of hepatocyte injury. Specifically, following two hours of reperfusion, AST and ALT levels in the I/R alone and the sild+I/R groups were significantly higher than in the sham and the sild+sham groups. However, sildenafil pretreatment attenuated hepatocellular injury, as the values of AST and ALT were significantly lower in the sild+I/R group, as compared to the I/R group (AST 222.7 ± 71.5 UI/dL versus 340.0 ± 67.2 UI/dL, *P* < 0.05, Figure 1) (ALT 326.9 ± 122.3 UI/dL versus 599.1 ± 137.2 UI/dL, Figure 2).

### 3.2. Histopathological Analysis

**Figure 3:**
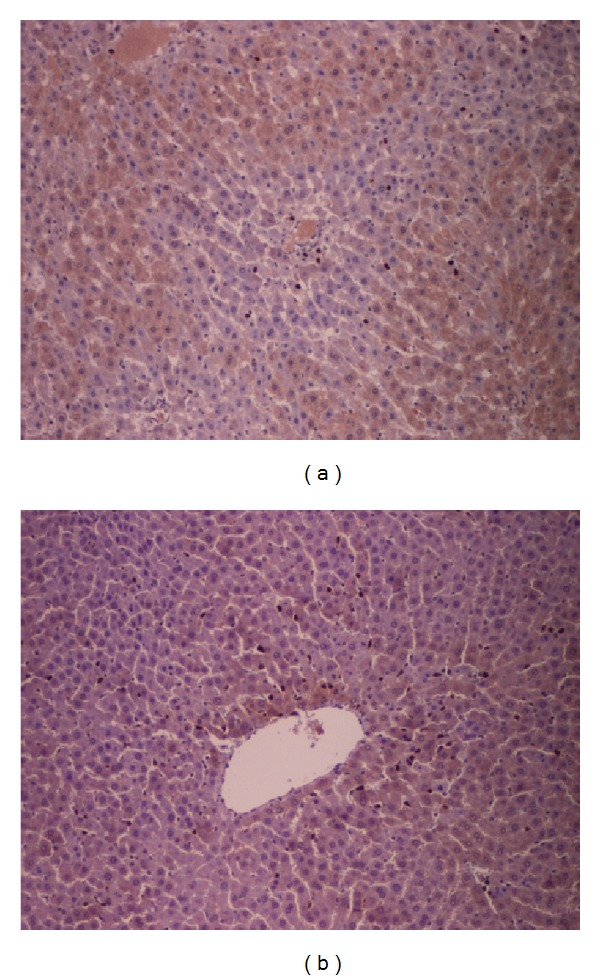
(a) Hepatocellular necrosis in zone 3 of the hepatic lobule and increased inflammatory infiltration in the I/R group (hematoxylin-eosin ×100). (b) Few foci of hepatocellular necrosis and mild inflammatory infiltration in the sild+I/R group (hematoxylin-eosin ×100).

Neither the sham group nor the sild+sham group showed any significant histological findings. Liver samples from the I/R group showed extensive areas of zone 3 necrosis. In addition, sinusoidal congestion, microthrombosis, eosinophilic degeneration with hepatocyte vacuolization, and neutrophil infiltration were present. Tissue samples from the sild+I/R group exhibited lower severity of lesions since foci of hepatocyte necrosis and leukocyte infiltration were markedly suppressed in comparison to the I/R group (Figures 3(a) and 3(b)). Morphological findings were further confirmed by the semiquantitative assessment, where the sild+I/R group had significantly lower scores of hepatocellular necrosis and inflammatory infiltration, as shown in Table 3.

### 3.3. Myeloperoxidase Staining

Animals subjected to liver I/R had increased expression of MPO activity in liver tissue compared to animals of the sham and sild+sham group and significantly higher expression of MPO activity as compared to animals of the sild+I/R group (*P* < 0.01) (Table 3).

### 3.4. Measurement of ICAM-1 mRNA Levels

**Figure 4:**
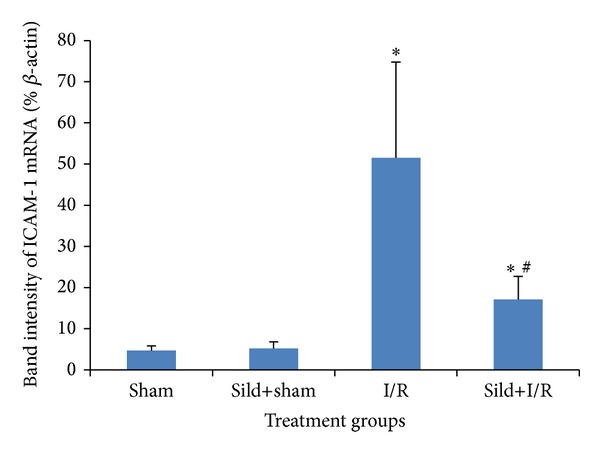
Band intensity of ICAM-1 mRNA as a percentage of *β*-actin; *P < 0.05 in comparison to the sham group; ^#^ *P* < 0.05 in comparison to the I/R group.

Through assessment of mRNA transcripts, liver I/R injury remarkably increased mRNA expression of ICAM-1, while sildenafil pretreatment in the sild+I/R group attenuated I/R-induced mRNA expression (Table 3, Figure 4).

### 3.5. Evaluation of Hepatic Apoptosis by TUNEL Staining

**Figure 5:**
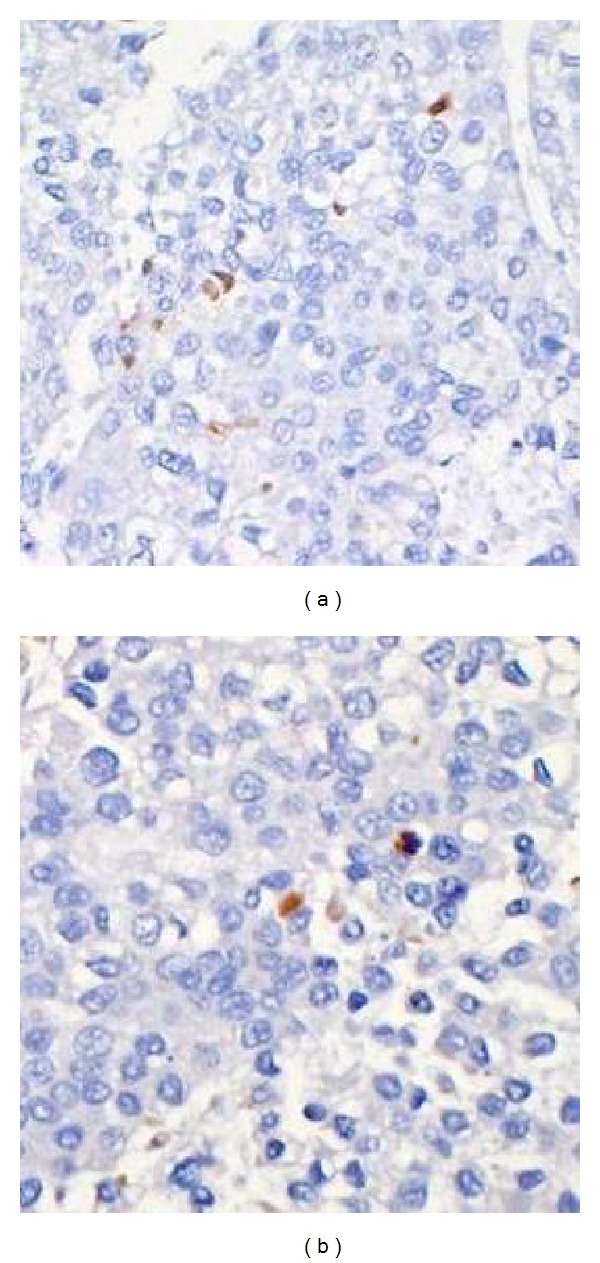
Apoptosis recorded as the percentage of positively staining nuclei per high-power field was higher in the I/R group (a) as compared to the sild+I/R group (b). TUNEL staining ×200, apoptotic bodies appear brown in color.

Specimens from the sild+I/R group showed significantly fewer cells stained positive by TUNEL versus the I/R group (32% ± 9% versus 58% ± 15% percentage of nuclei containing apoptotic bodies; *P* < 0.05) (Table 3, Figure 5).

## 4. Discussion

In the present study we used a partial liver ischemia-reperfusion model in rats in order to simulate various aspects of hepatic surgery under vascular occlusion, including surgery for liver tumors, liver transplantation, and hepatic trauma. Previous studies have shown that inflammation, apoptosis, and altered microcirculation are histological findings evident even in the early stage of hepatic ischemia-reperfusion injury.

The results of our study suggest that sildenafil used as pretreatment has a hepatoprotective effect in a rat model of partial liver ischemia-reperfusion injury. This was evidenced by suppression of the increase in AST and ALT, decreased scores of necrosis, attenuation of morphological liver injury, and antiapoptotic activity. Decreased expression of ICAM-1 mRNA and reduction of leukocyte-endothelial interaction, as evidenced by attenuated MPO staining in the sildenafil-treated rats, were also observed.

In the past, several studies have investigated the role of NO in partial liver ischemia-reperfusion models with controversial results [14]. Whether NO has a protective or deleterious effect probably depends on the type of insult, the source and quantity of NO produced, and the cellular redox status of the liver [16, 28–31]. It is considered that endogenous (basal) NO, produced by an early and transient activation of eNOS, protects both hepatocytes and endothelial cells against reperfusion injury in the liver [32]. NO counteracts the vasoconstriction caused by endothelin-1, which is involved in microvascular dysfunction, particularly during the early stages of liver I/R [33]. For instance, it has been found that I/R injury is exacerbated in e-NOS deficient animal models [34, 35], whereas genetic overexpression of eNOS has been shown to attenuate hepatic I/R injury in a rat model [36]. Therefore, eNOS expression has a cytoprotective effect by maintaining basal levels of NO production and acts protectively against the early phase of I/R injury by preservation of the sinusoidal structure and maintenance of blood flow through the hepatic microcirculation, thus limiting the extent of I/R injury through a cGMP pathway [14, 16]. eNOS expression is downregulated during liver reperfusion as a result of inhibition of eNOS activity by oxidative stress and absence of flow within the sinusoids during ischemia [14]. The decreased production of NO from eNOS increases the vascular resistance of the intrahepatic circulation and contributes to the microcirculatory failure following reperfusion [8, 10]. Analogous findings in the microcirculation have also been shown in the context of liver cirrhosis. In particular, a decrease of NO-related relaxation response or a reduction in hepatic NO bioavailability mediated via a decrease in eNOS activity seems to be one of the mechanisms leading to the increased intrahepatic resistance in cirrhotic livers [37]. Similarly, increased expression of PDE-5, which may enhance the degradation of hepatic cGMP and be involved in the decreased vasodilator response to NO, has been observed in the intrahepatic vasculature of cirrhotic rat livers [38].

On the other hand, the induction of iNOS, stimulated by oxidative stress during reperfusion and expressed within 6 hours of reperfusion, seems to increase reperfusion-mediated liver injury [16, 17, 39]. Once i-NOS is induced, excessive amounts of NO are produced. In the presence of superoxide, NO can form peroxynitrite, a potent oxidant and protein nitrating agent and a substance extremely toxic to cells. Peroxynitrite subsequently can decompose to generate a strong oxidant with reactivity similar to hydroxyl radical [40]. Therefore, apart from favorable actions, NO, produced in excess, may also prove hazardous and have cytotoxic potential through its interaction with superoxide anion and contribute to hepatic injury accompanying the late phases or reperfusion. Under normal conditions, excessive NO production clearance is achieved through hemoglobin. However, postreperfusion microcirculation failure may lead to impaired clearance, further enhancing the deleterious effects of NO during reperfusion.

Sildenafil is a potent inhibitor of PDE5, which catalyses the breakdown of cGMP. Thus, the administration of sildenafil preserves NO-driven cGMP levels by reducing its degradation and enhancing its action as a potent vasodilator and an inhibitor of thrombocyte aggregation [17, 19]. cGMP activates protein kinase G, which in turn opens mitochondrial adenosine triphosphate potassium (mitoK_ATP_) channels, conferring the protective effect against I/R injury [17]. Sildenafil has also been shown to upregulate eNOS expression, thus directly enhancing NO bioavailability [41, 42]. Moreover, there is substantial experimental evidence that activation of protein kinase C could be one of the intracellular signal transduction pathways controlling sildenafil-dependent cardiac protection in the rabbit heart [43]. A direct action of sildenafil on mitoK_ATP_ channels has also been suggested to mediate the sildenafil-induced protection against ischemic injury [44]. Additionally, sildenafil has been shown to have a protective effect independent of the NO/cGMP pathway [45]. Finally, a direct neutralization of free radicals has been attributed to sildenafil [46].

Sildenafil has been used in the past in experimental models of ischemia-reperfusion of various organs such as heart, kidney, brain, and lung [21–24]. It has also been administered in pulmonary hypertension settings with favorable outcomes [20, 47]. Literature is very scarce, if any, regarding the use of sildenafil in the setting of liver I/R injury.

In the present study we chose to administer sildenafil at a dose of 0.3 mg/kg. This dose corresponds to the dose administered in studies of pulmonary hypertension and was chosen because it has been shown to be well tolerated with no significant hemodynamic instability [25–27]. Intraperitoneal administration was preferred over* per os* administration due to the characteristic intestinal and hepatic first pass effect, which decreases bioavailability to 14.6% when administered orally [48]. The rationale for using sildenafil to attenuate I/R injury, despite the known deleterious effects of iNOS induced NO production during reperfusion, is that a selective PDE5 inhibitor, given as a pretreatment prior to ischemia and reperfusion, could enhance the beneficial action of NO during the reperfusion-induced suppression of eNOS production. In addition, sildenafil metabolism and elimination would prevent any enhancement on the late stage of reperfusion when iNOS induction has been shown to be cytostatic and antiproliferative.

According to our results, in animals pretreated with sildenafil before the onset of hepatic ischemia, attenuation of hepatocellular necrosis and inflammatory infiltration was noted in contrast to animals of the I/R group which exhibited a greater degree of necrosis and inflammation under microscopic evaluation. These findings delineate the beneficial effects of sildenafil pretreatment at the cellular level. Similarly, markers of hepatocyte injury, that is, AST and ALT, were attenuated in animals pretreated with sildenafil. This suggests that sildenafil may attenuate oxidative aggression-induced tissue injury through hepatocyte and sinusoidal endothelial cell membrane stabilization and minimization of intracellular calcium overload or through improvement of sinusoidal blood flow. The beneficial action of sildenafil administration in the microvascular level and the sinusoids has also been demonstrated in cirrhosis. In an experimental study, acute incubation of sildenafil increased the vasodilator response to NO in cirrhotic rat livers [38]. In a human study, acute administration of sildenafil increased hepatic production of NO and cGMP and decreased the hepatic sinusoid resistance in cirrhotic patients [49]. Chronic sildenafil administration also seems to have beneficial effects. In particular, in an animal study, one week of sildenafil treatment enhanced NO bioavailability and contributed to the attenuation of intrahepatic resistance in cirrhotic rat livers [50]. In that study, it was shown that the administration of sildenafil upregulated the hepatic protein expression of eNOS and increased intrahepatic NO production. Additionally, PDE-5 levels of sildenafil-treated cirrhotic livers were significantly reduced. Finally, a significant increase in sinusoid area indicating increased vasorelaxation of sinusoids and enhanced volumetric flow was demonstrated by the use of fluorescent microscopy and microcirculatory analysis. It was thus concluded that the NO/cGMP pathway is augmented in the hepatic microcirculation, which elicits vasorelaxation and increases intrahepatic blood flow [50]. Both the increased NO and inhibition of PDE-5 by sildenafil could contribute to the increase of hepatic cGMP levels. It has also been shown that high flow laminar shear stress increases production of NO though activation of eNOS [51, 52]. It can thus be postulated that the sildenafil-mediated increase of cGMP with the resulting increase in flow shear stress in the hepatic microcirculation contributes to the increased hepatic production of NO by sildenafil. The favorable effect of sildenafil in the microcirculation has also been demonstrated in the pulmonary and brain vasculature. Tantini et al. showed an antiproliferative effect of sildenafil on human pulmonary artery smooth muscle cells via inhibition of platelet derived growth factor-mediated activation of signal transduction pathways [53]. Additionally, Rosengarten et al. showed a beneficial effect of sildenafil on cerebral vascular reactivity [54]. Similar favorable vascular responses could underlie the improved histopathological and biochemical profile in animals pretreated with sildenafil before initiation of I/R in our experimental study. The hepatoprotection afforded by sildenafil could to a certain extent be also related to hepatoproliferative properties that have been attributed to sildenafil and its downstream signaling pathway. In particular, sildenafil promoted hepatocellular regeneration in a rat model of liver injury caused by chronic ethanol feeding, manifested by a greater mitotic index of liver cells in sildenafil-treated rats [55]. In addition, sildenafil administration in a rat model of partial hepatectomy accelerated regeneration rate of the remnant mass, as expressed by cell proliferation markers and mitotic counts [56]. The beneficial effects of sildenafil on hepatic regeneration at the cellular level have recently been demonstrated in a paracetamol-induced hepatotoxicity rat model [57].

Moreover, MPO staining in the sild-I/R group was significantly attenuated as compared to the nonsildenafil-treated animals. MPO is a neutrophil-specific enzyme, which is secreted during polymorphonuclear accumulation and is used as an index of hepatic leukocyte infiltration [58, 59]. As already mentioned, during ischemia, neutrophils accumulate in the endothelium and such accumulation may be markedly accelerated following reperfusion. Activated neutrophils release a variety of cytotoxic substances interacting with the endothelium and thereby causing tissue damage [60]. Moreover, aggregated neutrophils can physically obstruct capillary flow, causing further ischemia of the tissue and, lastly, they release large amounts of ROS, which contribute to the oxidative injury associated with hepatic I/R injury [61]. Therefore, as MPO activity is directly proportional to the neutrophil count, reduced MPO staining in the sildenafil-pretreated animals could be attributed to the attenuated neutrophil migration and activation during reperfusion.

Decreased expression of ICAM-1 mRNA with sildenafil administration was also observed. ICAM-1 is one of the adhesion molecules that are known to play an important role in the process of leukocyte-endothelial cell interaction. The adhesion of leukocytes to the microvascular endothelium is a manifestation of I/R injury and is mediated by a variety of cell-surface molecules like ICAM-1. This molecule is present at low levels on most endothelial cells and is upregulated in case of inflammation and I/R injury [10, 62]. In fact, blocking its activity with monoclonal antibodies has been found to protect against I/R injury [63, 64]. Moreover, NO donors have been found to attenuate leukocyte-endothelial cell reaction and to minimize the adhesive interactions between leukocytes and the endothelial cell surface, thus maintaining vascular patency [65–67]. Therefore, it is possible that sildenafil, through its NO downstream pathway, minimizes those adhesive interactions between leukocytes and the endothelial cell surface and, together with suppression of neutrophil tissue migration and infiltration, as shown with the attenuation of MPO activity, preserves vascular permeability and improves hepatic microcirculation.

Regarding apoptotic response, the sildenafil-pretreated group showed significantly fewer cells positive to TUNEL staining in comparison to the animals of the I/R only group. Although an excessive inflammatory response and necrosis are widely considered to be the major characteristic in the process of liver damage after I/R injury, there is also evidence that cell apoptosis could also be a primary mechanism of damage [11, 12, 68]. It could be possible that some of the protective effects elicited by sildenafil could be conferred through modulation of the apoptotic response. Sildenafil has been shown to directly protect adult cardiomyocytes against apoptosis following I/R injury in mice models [42, 69, 70]. It has also been shown that physiologically stimulated by NO soluble guanylate cyclase inhibits apoptosis [71]. Another study has suggested that eNOS-derived NO might assist in decreasing the percentage of apoptotic cells during hypoxia/reoxygenation in a lung model [72]. In addition, a report by Akao et al. has shown that openers of the mitoK_ATP_ channel were able to reduce apoptosis induced by oxidative stress in neonatal rat cardiomyocytes [73]. Therefore, we could hypothesize that a NO-cGMP signaling pathway could underlie the protective effect of sildenafil against hepatic apoptosis through its mitoK_ATP_ channel opening properties, as this has already been demonstrated in a rabbit heart model [44]. Our study is the first to demonstrate an antiapoptotic effect of sildenafil in experimental liver I/R injury through TUNEL staining. Further studies are required though to delineate the exact antiapoptotic pathways involved in sildenafil action.

Our study carries certain limitations, including the brief monitoring period as well as the fact that there was no hemodynamic monitoring of the animals during our experiment. Moreover, we studied a single dose of sildenafil, so we can only speculate about effect of different dosing regimens.

In conclusion, the results of this preliminary report suggest that sildenafil seems to attenuate hepatic ischemia-reperfusion injury when administered intraperitoneally prior to liver ischemia according to morphological and functional criteria, with preischemic administration mimicking the physiological phenomenon of preconditioning. This concept needs to be further investigated using different dosage schemes, administration times, and duration of treatment in order to extrapolate the results of this experimental study to realistic clinical environments and in order to be able to evaluate the sildenafil-mediated protection of hepatocytes during liver transplantation and in the setting of major liver resections, where metabolic and energy support of a small liver remnant could be of great importance. In addition, sildenafil administration could also be investigated in a posttreatment fashion, since there may be clinical occurrences where the onset of reperfusion is more predictable and it may be more feasible to initiate administration at reperfusion rather than before the ischemic event. Finally, future studies should include hemodynamic monitoring of sildenafil administration in the setting of liver I/R injury and also evaluate the exact mechanism of sildenafil action on apoptosis pathways.

## Figures and Tables

**Table 1:** Liver pathology scoring.

Hepatocellular necrosis	
<2 foci in every hepatic lobule	1
3–5 foci in every hepatic lobule	2
<5 foci in every hepatic lobule	3
Diffuse necrosis in zone 3	4
Presence of bridging necrosis	5
Extensive necrosis	6
Inflammatory infiltration of portal spaces	
Mild inflammation	1
Moderate inflammation	2
Severe inflammation	3

**Table 2:** Quantification of myeloperoxidase staining.

Positive cells for myeloperoxidase staining per three optic fields (40x magnification)	Score
0–20 cells	0
20–40 cells	1
40–60 cells	2
>60 cells	3

**Table 3:** Effect of sildenafil pretreatment on histopathology scores, MPO staining, ICAM-1 mRNA levels (as a percentage of *β*-actin), and percentage of apoptotic bodies.

	Sham group (*n* = 10)	Sild+sham group (*n* = 10)	I/R group (*n* = 10)	Sild+I/R group (*n* = 10)
Histology				
Hepatocellular necrosis scoring Inflammatory infiltration scoring	1 [1-1] 1 [1-1]	1 [1-1] 1 [1-1]	5 [5-6]* 3 [3-3]*	2.5 [2-3]^∗#^ 2 [1-2]^∗#^
MPO staining scoring	0 [0-0]	0 [0-1]	3 [2-3]*	1 [1-1]^∗#^
ICAM-1 mRNA levels (% of *β*-actin)	4.7 ± 1.1	5.2 ± 1.6	51.5 ± 23.3*	17.1 ± 5.6^∗#^
Apoptotic bodies (%)	14 ± 5	15 ± 7	58 ± 15*	32 ± 9^∗#^

Values are presented as mean ± SD for ICAM-1 mRNA levels and apoptotic bodies and as median [25th–75th percentiles] for histology and MPO staining scores.

*P < 0.05 in comparison to the sham group; ^#^ *P* < 0.05 in comparison to the I/R group.
